# Lessons from the pandemic and the value of a structured system of ultrasonographic findings in the diagnosis of COVID-19 pulmonary manifestations

**DOI:** 10.31744/einstein_journal/2024AE0780

**Published:** 2024-05-20

**Authors:** Vítor Carminatti Romano, Natália Tavares de Melo Barros Lima, Victor Arantes Jabour, Guilherme Ciconelli Del Guerra, Paulo Rogério Barboza Silvério, Rodrigo Gobbo Garcia, Yoshino Tamaki Sameshima, Miguel José Francisco Neto, Marcos Roberto Gomes de Queiroz

**Affiliations:** 1 Hospital Israelita Albert Einstein São Paulo SP Brazil Hospital Israelita Albert Einstein, São Paulo, SP, Brazil.

**Keywords:** COVID-19, Coronavirus infections, SARS-CoV-2, Ultrasonography, Lung disease, Clinical decision making, Organ dysfunction

## Abstract

**Objective:**

We aimed to share our experience in implementing a structured system for COVID-19 lung findings, elucidating key aspects of the lung ultrasound score to facilitate its standardized clinical use beyond the pandemic scenario.

**Methods:**

Using a scoring system to classify the extent of lung involvement, we retrospectively analyzed the ultrasound reports performed in our institution according to COVID-RADS standardization.

**Results:**

The study included 69 thoracic ultrasound exams, with 27 following the protocol. The majority of patients were female (52%), with ages ranging from 1 to 96 years and an average of 56 years. Classification according to COVID-RADS was as follows: 11.1% in category 0, 37% in category 1, 44.4% in category 2, and 7.4% in category 3. Ground-glass opacities on tomography correlated with higher COVID-RADS scores (categories 2 and 3) in 82% of cases. Ventilatory assessment revealed that 50% of cases in higher COVID-RADS categories (2 and 3) required second-line oxygen supplementation, while none of the cases in lower categories (0 and 1) utilized this support.

**Conclusion:**

Lung ultrasound has been widely utilized as a diagnostic tool owing to its availability and simplicity of application. In the context of the pandemic emergency, a pressing need for a focused and easily applicable assessment arose. The structured reporting system, incorporating ultrasound findings for stratification, demonstrated ease of replicability. This system stands as a crucial tool for screening, predicting severity, and aiding in medical decisions, even in a non-pandemic context.

## In Brief

Implementing a structured COVID-19 lung ultrasound system, using COVID-RADS standardization. This case series exams revealed correlations between ultrasonographic and tomographic findings. Ventilatory assessments showed that higher categories required second-line oxygen. This replicable tool will aid in screening and predicting disease severity beyond the pandemic.

## Highlights

Lung ultrasound enables precise diagnosis and ongoing monitoring of the disease.

Ultrasound is an effective tool for assessing pulmonary findings in COVID-19.

Structured reports enhance communication and are easily reproducible.

## Graphical abstract

**Figure:**
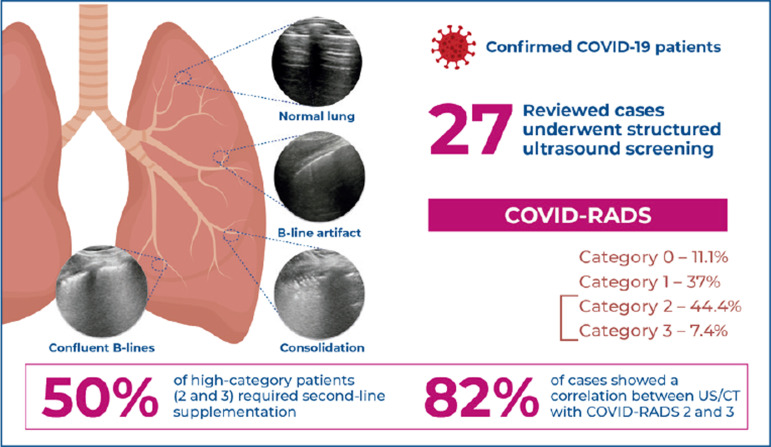

## INTRODUCTION

The COVID-19 pandemic triggered by the SARS-CoV-2 coronavirus reached global proportions in the initial months of 2020. Notably, higher fatality rates were observed among individuals aged 60 and above, as well as among those with underlying comorbidities.

In this context, it is imperative to explore examination methodologies that can be deployed on a large scale to ensure cost-effectiveness and the possibility of being conducted at the patient’s bedside with minimal or reduced side effects. This consideration extends to the training of clinical and generalist physicians engaged in pandemic response activities in general hospitals, intensive care units, and field hospitals.

The pulmonary manifestations observed in patients infected with SARS-CoV-2 variants remain the most concerning and aggressive, often exhibiting rapid progression from symptom onset. A wide spectrum of findings exists within this range of manifestations, ranging from milder forms of involvement to striking patterns requiring orotracheal intubation.

Early identification of these imaging findings, as well as the stratification of lung involvement, can greatly assist in both the rapid diagnosis and close monitoring of patients at a higher risk of unfavorable outcomes. Similarly, targeted pulmonary imaging examinations allow for monitoring and quantification of the response pattern to the proposed treatments.

**Figure 1:**
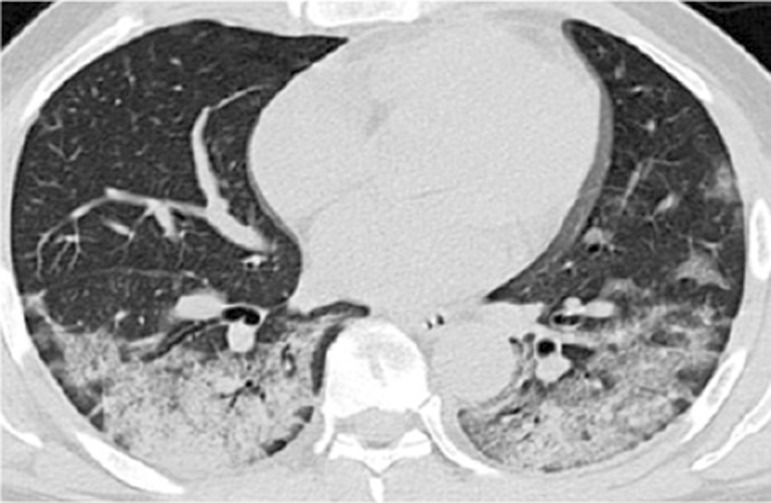
Typical computed tomography findings of COVID-19 pneumonia: scattered multifocal and bilateral ground-glass opacities in the lungs, predominantly in the peripheral and posterior regions

Initial reports of pulmonary manifestations studied by computed tomography in this condition described peripheral ground-glass opacities, predominantly in the posterior lung segments, as well as centrilobular consolidations and mosaic patterns.^(1,2)^ Chest radiography is definitively limited for this evaluation because it lacks adequate sensitivity for detecting ground-glass patterns (Figure 1).

Given the limitations of widespread computed tomography use owing to transportation difficulties and high costs, ultrasound is a prominent tool because of its low cost, accessibility, mobility, and potential for protocols that facilitate replicability in point-of-care training. Therefore, it is important to study the potential of ultrasound in relation to the pulmonary findings of this viral pathology. In this context, we understand that it is important to document the lessons learned from the application of this method for recording and mapping future studies on viral etiology and pulmonary pathologies that may present similar findings.

Ultrasound examination is one of the main diagnostic assessments for various clinical conditions because of its quick execution. Given the need for early diagnosis of acute respiratory pathologies and minimizing potential complications due to delayed treatment, it stands out as a method for evaluating dyspnea and acute respiratory failure. The absence of ionizing radiation is a significant advantage of this modality, both for physicians and patients who will undergo evaluation, as well as for the entire healthcare team of a given hospital unit.

The possibility of establishing a scoring system for ultrasound findings with increasing degrees of complexity, similar to what occurs in thyroid, breast, prostate, and hepatic evaluations, is another important advantage in a global health emergency context. This will enable the development of care protocols and future research using multicenter and standardized protocols.^(3)^

Based on existing evidence and records, ultrasound is an effective and robust examination method for assessing lung conditions of different etiologies, playing an important role in diagnosing the causal factors of respiratory failure. It has also been widely used for structured reports in recent fields, such as appendicular evaluation in emergencies and in the study of patients with shock and cardiac arrest.^(4-10)^

Considering that COVID-19 is a recent infection in our environment, with many uncertainties surrounding the virological characteristics and systemic responses in different age and racial groups, post-pandemic bibliographic data on the use of ultrasound in pulmonary assessments have generated other structured systems for the use of pulmonary ultrasound in patients infected with this virus. Its application has proven useful, reinforcing the importance of reporting various experiences with this methodology to advance pulmonary ultrasound and enhance its scientific maturation in advanced care settings.

## OBJECTIVE

We aimed to share our experience in implementing a structured system for COVID-19 lung findings, elucidating key aspects of the lung ultrasound score to facilitate its standardized clinical use beyond the pandemic scenario.

## METHODS

To evaluate the suitability of chest ultrasound for diagnosing and monitoring pulmonary issues related to COVID-19, a retrospective analysis was conducted on ultrasound reports from patients treated at *Hospital Israelita Albert Einstein*, covering several units within the institution.

Examinations were performed using Philips IU 22, Epic 7, and Logic 9 ultrasound equipment with linear (5–12 MHz) and convex (3–6 MHz) transducers. Accessible lung parenchyma was evaluated using B-mode and Doppler techniques by experienced physicians from the Ultrasonography Team of the Imaging Department at *Hospital Israelita Albert Einstein*, all of whom had more than four years of ultrasound experience and were well acquainted with conducting lung ultrasounds.

The study project was approved by the ethics committee of the *Hospital Israelita Albert Einstein* institution, (CAAE: 35861020.4.0000.0071; #165.746).

### Study design

This study adopted a case series approach. Patient records were collected, including ultrasound reports, computed tomography reports conducted within four days of the ultrasound, and clinical data, such as oxygen saturation and oxygen supplementation details, within two days of the ultrasound.

Sonographic findings were described and organized according to the tables provided (Appendices 1 and 2) and categorized from P1 to P4, with 4 further divided into P4A and P4B. A specific region of interest could display multiple sonographic anomalies, with the highest score dictating the classification of that region (*e.g*., region 1, score 2; region 2, score 1, and so on) (Appendix 3). The cumulative score for each studied region was then used for classification according to COVID-RADS (Appendix 4).

Appropriate personal protective materials and equipment, as well as attire adhering to the institutional hygiene guidelines, were used (Appendix 5).

**Figure 2:**
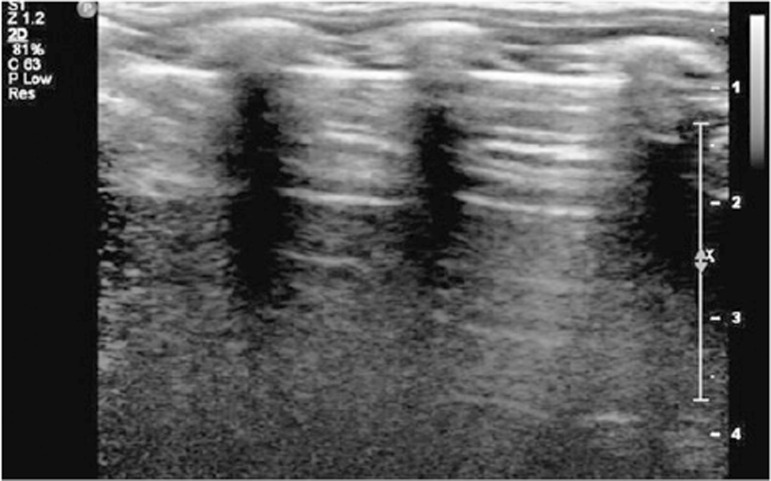
Ultrasonographic A-line artifacts: horizontal lines equidistant and parallel to the pleural line

**Figure 3:**
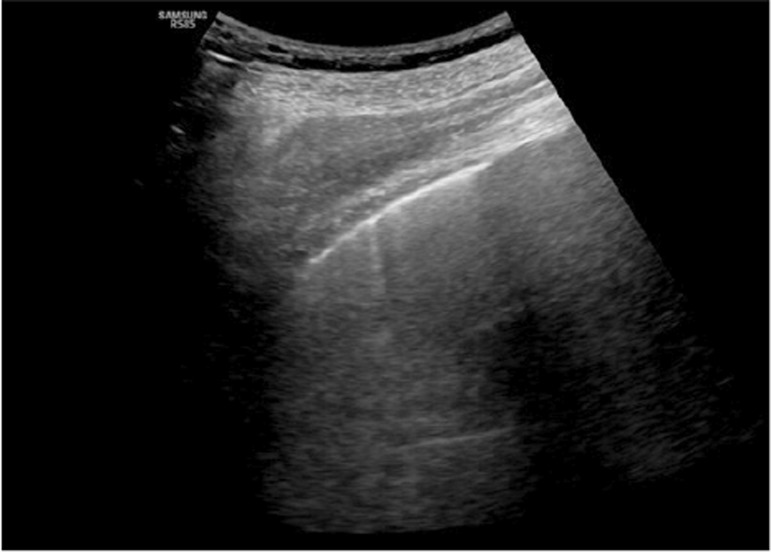
Ultrasonographic B-line artifacts: vertical lines moving in conjunction with the lung sliding from the pleural line to the screen edge and erasing the A-lines

**Figure 4:**
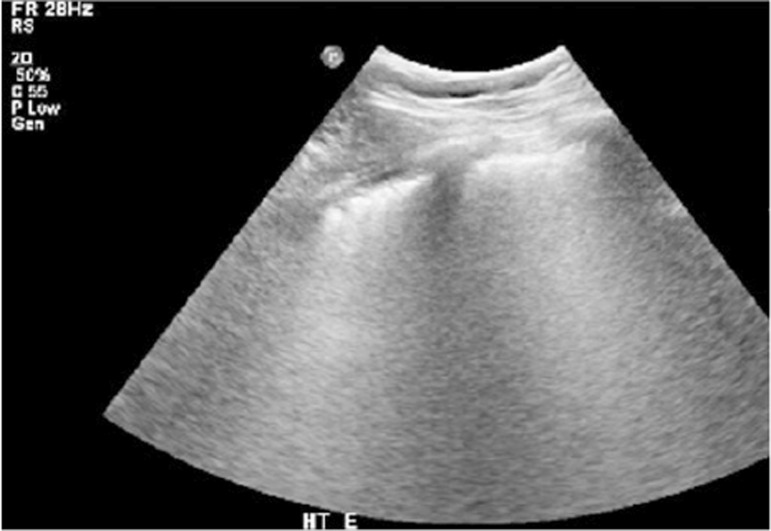
Ultrasonographic ground-glass: multiple confluent B-lines corresponding to the “ground glass” pattern on computed tomography

Ultrasound findings were divided into four categories, as previously outlined. The pleural line was defined as a horizontal hyperechoic line situated approximately 0.5cm below the rib. Horizontal lines equidistant and parallel to the pleural line were termed A-lines (Figure 2), while vertical lines moving in conjunction with the lung sliding from the pleural line to the screen edge and erasing the A-lines were termed B-lines or comet tails (Figure 3). An increasing number of B-lines corresponded to a more severe interstitial pathology, indicating a shift from moderate to complete loss of aeration. If the number of B-lines exceeded three or converged, a “white lung” appearance was observed (Figure 4), often correlated with ground-glass opacities on computed tomography (Figure 1).^(11-13)^

Pleural effusion refers to a hypoechoic or anechoic collection between the parietal and visceral pleura. When observed using M Mode, a “sinusoidal signal” could be seen due to the movement of the lung within the fluid pleural effusion. Transudates typically appear homogeneous and anechoic, whereas exudates may exhibit heterogeneity and loculation.^(11)^

**Figure 5:**
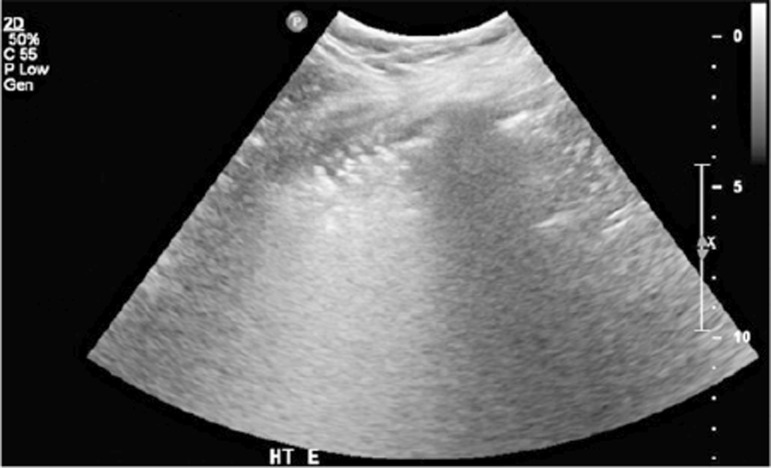
Lung consolidation: multiple hyperechogenic foci (air bronchograms) surrounded by a hypoechogenic area

Alveolar consolidation was characterized by hypoechogenic ill-defined areas within one or more lung regions, accompanied by mobile hyperechogenic foci during breathing (air bronchograms) (Figure 5).^(12,13)^

The tomographic analysis considered the degree of involvement by underlying pathology, which typically manifests as ground-glass opacities,^(1,2)^ and was graded as <25%, 25–50%, close to 50%, or >50%.

### Population

#### Inclusion criteria

This study included patients diagnosed with COVID-19 through laboratory testing who presented with clinical indications of respiratory symptoms across varying degrees of severity and who underwent thoracic ultrasound. All examinations were conducted between April and December 2020.

#### Exclusion criteria

Patients with pre-existing chronic lung diseases, primary or metastatic lung tumors, and pneumonia stemming from other etiologies, especially non-viral causes, were excluded from the study.

## RESULTS

Of the 69 thoracic ultrasound examinations, 27 followed the protocol. The patients’ data are presented in table 1. The majority of the population consisted of women (52%), with ages ranging from 1 to 96 years and an average age of 56 years. The classification according to COVID-RADS was distributed as follows: three cases (11.1%) in category 0, 10 cases (37%) in category 1, 12 cases (44.4%) in category 2, and two cases (7.4%) in category 3.

**Table 1:** Patient demographics

	Age	Gender	Laterality	Sum	COVID-RADS	Ground- glass (CT) (%)	O_2_ Saturation (%)	O_2_ Support
1	77	M	9 (R) 9 (L)	18	1	25–50	>93	AA
2	60	F	6 (R) 6 (L)	12	0	0	>95	AA
3	54	M	9 (R) 9 (L)	18	1	25–50	100	AA
4	75	M	17 (R) 14 (L)	31	2	>50	95	NRM 8L/min
5	44	F	10 (R) 14 (L)	24	2	NR	96	AA
6	25	M	15 (R) 14 (L)	29	2	NR	92	NC 3L/min
7	73	M	21 (R) 21 (L)	42	3	~50	>95	NC 3L/min
8	69	M	17 (R) 15 (L)	32	2	25–50	94	NC 2L/min
9	64	M	12 (R) 13 (L)	25	2	~50	>96	AA
10	81	F	14 (R) 17 (L)	31	2	25–50	92	NC 1L/min
11	62	F	13 (R) 12 (L)	25	2	>50	92	NRM 8L/min
12	68	M	16 (R) 15 (L)	31	2	>50	90	MV OTT FiO2 40% 40L/min
13	49	F	18 (R) 19 (L)	37	2	>>50	91	HFNC FiO2 80%
14	1	M	6 (R) 6 (L)	12	0	NR	97	AA
15	55	F	6 (R) 6 (L)	12	0	NR	No data	
16	27	F	7 (R) 7 (L)	14	1	NR	98	AA
17	45	M	8 (R) 10 (L)	18	1	<25	No data	
18	60	M	8 (R) 11 (L)	19	1	~50	95	AA
19	48	F	6 (R) 10 (L)	16	1	<25	96	AA
20	33	F	7 (R) 7 (L)	14	1	<25	99	AA
21	96	F	15 (R) 18 (L)	33	2	>50	91	BiPap FiO2 40%
22	96	F	20 (R) 18 (L)	38	2	>50	92	MV OTT FiO2 40%
23	86	M	18 (R) 24 (L)	42	3	>50	94	MV OTT FiO2 40%
24	39	F	6 (R) 6 (L)	12	1	NR	98	AA
25	12	F	6 (R) 6 (L)	12	1	NR	99	AA
26	39	F	16 (R) 17 (L)	33	2	NR	94	NC 2L/min
27	82	M	7 (R) 9 (L)	16	1	25–50	95	NC 2L/min

CT: computed tomography; NR: not performed; AA: ambient air; NC: nasal catheter; NRM: non-rebreathing mask; BiPap: bilevel positive airway pressure; HFNC: high-flow nasal catheter; MV OTT: mechanical ventilation with an orotracheal tube.

The tomographic findings of ground-glass opacities were distributed based on the degree of lung involvement as follows: for cases with higher COVID-RADS scores (categories 2 and 3), there was a correlation with the degree of ground-glass opacities on tomography (≥50%) in 82% (9/11) of the cases. Among cases classified as 0 and 1, only one case, among those who underwent tomography within the recommended interval, had an involvement degree close to 50%, with all other cases showing less than 50% involvement.

The ventilatory status was assessed based on the degree of peripheral oxygen saturation using pulse oximetry and its correlation with ventilatory support. Among the cases with higher COVID-RADS scores (categories 2 and 3), 50% (7/14) required second-line oxygen supplementation (non-rebreather mask, high-flow nasal cannula, and non-invasive and invasive ventilation). However, second-line oxygen supplementation was not used in any of the cases in categories 0 and 1.

## DISCUSSION

In response to the global COVID-19 pandemic caused by the SARS-CoV-2 Coronavirus, the necessity for accessible and effective diagnostic methodologies has become increasingly apparent. Given the aggressive nature of respiratory manifestations, the assessment of pulmonary findings has taken center stage. In this context, our research project was designed to explore the potential of ultrasound as a viable alternative for evaluating the lungs of patients with COVID-19.

This retrospective observational study examined patients diagnosed with COVID-19 who presented with respiratory symptoms. A team of experienced professionals performed ultrasonography using specialized equipment. The ultrasound findings were systematically categorized into grades ranging from 0 to 3, representing distinct lung imaging patterns.

Our analysis revealed that ultrasonography is an effective tool for assessing pulmonary findings in patients with COVID-19. Categorization of these findings into degrees allowed for systematic stratification that correlated with disease severity. The use of structured reports has emerged as a promising methodology, enhancing communication among healthcare professionals and proving to be both easily reproducible and conducive to streamlining therapeutic decisions.

Notably, leveraging the increasingly detailed knowledge of pulmonary findings during the coronavirus pandemic is crucial. The use of ultrasound as a diagnostic tool enables precise diagnosis and ongoing monitoring of the disease. Ultrasound has emerged as a highly valued method and may even become the primary imaging method for patients with this condition.^(14-21)^

The limitations of computed tomography in specific situations, such as rapid disease progression or limited availability,^(21)^ underscore the importance of ultrasonography as a dynamic, accessible, and cost-effective approach. Its advantages include the absence of ionizing radiation, mobility, and the potential for large-scale training.

In this context, a structured reporting system should be considered to enhance the consistency and clarity of communication among healthcare professionals, thereby ensuring the effective utilization of ultrasound in the diagnosis and management of COVID-19.

## Figures and Tables

**Figure:**
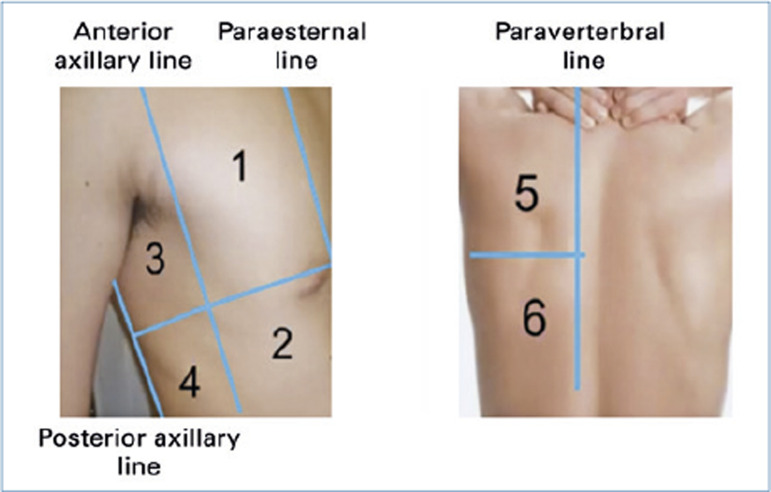
Enumeration of the thoracic regions for systematic analysis Ech lung field within these regions should be scored independently. The regions were numbered from 1 to 6 on both sides of the chest, ventrally and dorsally.

## Appendix 2

**Table:** The six lung regions to be examined were numbered from 1 to 6 and scored from 1 to 5

	Region	Score
Anterosuperior	R	1 2 3 4 5
Anteroinferior	R	1 2 3 4 5
Lateral superior	R	1 2 3 4 5
Lateral inferior	R	1 2 3 4 5
Posterosuperior	R	1 2 3 4 5
Posteroinferior	R	1 2 3 4 5
Anterosuperior	L	1 2 3 4 5
Anteroinferior	L	1 2 3 4 5
Lateral superior	L	1 2 3 4 5
Lateral inferior	L	1 2 3 4 5
Posterosuperior	L	1 2 3 4 5
Posteroinferior	L	1 2 3 4 5

R: right; L: left.

## Appendix 3

**Table:** Ultrasonographic findings in COVID-19 lung disease

P1) Normal aeration (pleural sliding present, normal pleural line echo, A-lines, and up to 2 B-lines).	P1 - 1 point
P2) Interstitial pattern (distinct and well-defined B-lines >3 per field); thickening (greater than 1 mm) and irregularity of the pleural line echo.	P2 - 2 points
P3) Ground-glass opacities (confluent B-lines).	P3 - 3 points
P4) Pulmonary consolidation (hypoechogenic area with air/fluid bronchograms) P4A - Consolidation <2.5cm. P4B - Consolidation >2.5cm.	P4A - 4 points P4B - 5 points

The final score of the pulmonary ultrasound, ranging from 6 to 60, was calculated as the sum of points (from the lowest possible score of 1 to the highest score of 5 in each field).

## Appendix 4

**Table:** COVID-RADS: Structured ultrasound classification summary

COVID-RADS	Findings	Points
0	Normal findings	<12
1	Minor findings	13–19
2	Confluent B-lines (ground-glass) multifocal	20–39
3	Major findings: Confluent B-lines (ground-glass) and consolidations	≥ 40

## Appendix 5

**Table:** Contamination control routine to perform the exam

A) Before entering the room: •Clean your machine with appropriate products •Be thorough •Remove anything unnecessary •Set the presets in advance •Use individual gel containers that will be disposed of •Dress according to the protocol •Prepare an ultrasound cleaning kit consisting of an antiseptic solution, cleaning wipes, and an extra pair of gloves •Ask for assistance to open the door, and enter with the equipment B) Inside the room: •Clean your hands, put on gloves, cover the transducers, and perform examinations •At the end, still wearing personal protective equipment (PPE), remove gloves, clean your hands, and put on a new pair of gloves •Using an appropriate cloth, dampen all surfaces of the machine, including cables, keyboards, screens, and transducers (non-disposable), as well as cables, power cords, and gel containers (disposable) •Check for splashes •Clean crevices and containers •Complete the room phase of the doffing protocol •With one hand, open the door and use the other hand to remove the equipment from the room C) After leaving the room: •Complete the room phase of the doffing protocol •Inspect the machine for splashes and droplets •Clean again if necessary •Wait for the product to dry
